# Evidence for maintenance of key components of vocal learning in ageing budgerigars despite diminished affiliative social interaction

**DOI:** 10.1098/rspb.2023.0365

**Published:** 2023-06-14

**Authors:** Bushra Moussaoui, Samantha L. Overcashier, Gregory M. Kohn, Marcelo Araya-Salas, Timothy F. Wright

**Affiliations:** ^1^ Department of Biology, New Mexico State University, Las Cruces, NM 88003, USA; ^2^ Department of Psychology, University of North Florida, Jacksonville, FL 32224, USA; ^3^ Centro de Investigación en Neurociencias & Escuela de Biología, Universidad de Costa Rica, San Pedro, San José, Costa Rica

**Keywords:** ageing, budgerigars, vocal learning, cognitive senescence, social networks

## Abstract

In some species, the ability to acquire new vocalizations persists into adulthood and may be an important mediator of social interactions. While it is generally assumed that vocal learning persists undiminished throughout the lifespan of these open-ended learners, the stability of this trait remains largely unexplored. We hypothesize that vocal learning exhibits senescence, as is typical of complex cognitive traits, and that this decline relates to age-dependent changes in social behaviour. The budgerigar (*Melopsittacus undulatus*), an open-ended learner that develops new contact call types that are shared with social associates upon joining new flocks, provides a robust assay for measuring the effects of ageing on vocal learning ability. We formed captive flocks of 4 previously unfamiliar adult males of the same age class, either ‘young adults’ (6 mo−1 y) or ‘older adults’ (≥ 3 y), and concurrently tracked changes in contact call structure and social interactions over time. Older adults exhibited decreased vocal diversity, which may be related to sparser and weaker affiliative bonds observed in older adults. Older adults, however, displayed equivalent levels of vocal plasticity and vocal convergence compared to young adults, suggesting that many components of vocal learning are largely maintained into later adulthood in an open-ended learner.

## 1. Introduction

Ageing is accompanied by the progressive decline, or senescence, of vital physiological and behavioural functions [1]. Senescence is particularly well characterized in humans, where it includes diminished cognition and motor performance [2,3], increased risk for certain pathologies such as cancer, diabetes and neurodegenerative diseases [4], as well as changes in social relationships [5,6]. Further contributing to the reduced social well-being of some ageing adults are cognitive declines in language proficiency related to impaired speech production, reduced working memory capacity and slower sensory/perceptive processing speeds [7]. Although language learning primarily occurs during childhood, learning of new words, regional dialects and even additional languages continues into adulthood. This flexibility in speech and language learning capacity may be particularly important for certain adult populations such as immigrants, aphasia patients and cochlear implant recipients [8,9].

While language is unique to humans, several distantly related animal taxa (e.g. elephants, cetaceans, pinnipeds, bats, songbirds, parrots and hummingbirds) exhibit the ability to imitate sounds; this trait is known as vocal production learning (hereafter ‘vocal learning’) and is a key component of language [10]. The identification of numerous parallels between human speech learning and avian vocal learning, including a dependence on feedback from conspecific adult tutors, parallel neuroarchitectures and convergent gene expression patterns, have made vocal learning birds like songbirds and parrots popular models for investigating the biological underpinnings of this complex cognitive trait [11,12]. While many songbirds are restricted to an early life learning period and classified as closed-ended vocal learners [13], most parrots, including the commonly studied budgerigar, *Melopsittacus undulatus*, are considered open-ended learners that continue producing new vocalizations as adults [14,15]. Such open-ended learners provide a useful model for studying the effects of ageing on vocal learning.

Although it is generally assumed that vocal learning persists unabated throughout the lifespan of open-ended learners, the extent to which this is true remains largely unexplored. Most studies on ageing and vocalizations have been in closed-ended songbird models and focus on vocal performance traits such as pitch, amplitude and stereotypy rather than learning [16,17]. One exception is a study on age-associated changes in song traits of adult female European starlings (*Sturnus vulgaris*), which found that while song repertoire size decreased with age, song sharing rates were higher in older birds [18]. It remains unclear whether higher song sharing in older adults can be attributed to active vocal learning or a selective pruning of the vocal repertoire as individuals age.

Age-associated changes in vocal learning ability may also impact the quality of social relationships, given the intimate link between vocal learning and communication. New vocalizations are typically acquired via imitation of and feedback from social partners [12], and in some species, learned vocalizations are used to mediate complex social relationships by encoding individual identity, signalling group membership, or conveying local knowledge [19]. Thus, a complementary investigation of age-related changes in social behaviour can help elucidate the relationship between vocal learning ability and sociality, or the degree to which animals interact and form associations. Studies in humans and non-human primates have generally identified decreased social interactions, smaller social networks and social selectivity as defining features of ageing [5,20]. For instance, social selectivity exhibited by older male chimpanzees (*Pan troglodytes*) involves engaging in more mutual friendships, exhibiting a bias towards positive interactions and preferentially socializing with important individuals [5,21]. Similarly, ageing tufted capuchin monkeys (*Sapajus* sp.) increasingly direct grooming to a single preferred partner and exhibit a decreased affiliative social interaction frequency [22]. Social ageing remains largely unexplored in avian taxa, with the exception of a recent study in sulfur-crested cockatoos (*Cacatua galerita*) that found an increase in social stability with age and stronger social associations in juveniles than adults, albeit without an age-related difference in total number of associations [23]. It is unclear, however, if this particular age-associated pattern in social dynamics represents a generalizable avian social ageing phenotype since social ageing has not been directly investigated in other avian taxa.

Parrots (order Psittaciformes) are particularly long-lived and highly social open-ended learners. They are well known for their extensive vocal mimicry capabilities, which allow them to imitate human speech in captivity, and amass large vocal repertoires in the wild [24]. Evidence suggests that the widespread ability to mimic conspecific calls facilitates social interactions in fission–fusion dynamics of many parrot species [25]. The budgerigar, a gregarious flock-living parakeet native to Australia, is especially well suited for investigating the relationship between ageing and vocal learning in a rich social context. The contact call, produced to coordinate behaviour when birds are separated from flockmates, is the most commonly used learned vocalization [26]. Individuals of both sexes have a repertoire of several contact call types, characterized by unique frequency modulation patterns, that are shared with members of their social groups [27]. Captive experimental manipulations of social contexts have shown that male budgerigars imitate the contact calls of female mates [15], and that in response to joining new social groups, budgerigars of both sexes converge on shared contact calls at multiple social levels [14,28,29]. In addition to its amenability to captive assays of vocal learning, the budgerigar is long-lived, with a maximum recorded lifespan in captivity of 18.01 years [30] and mean life expectancy of 4.57 years [31], providing ample opportunities for sampling vocal learning at many ages.

Here we test whether vocal learning diminishes with advanced age in an open-ended vocal learner and how this socially mediated trait may be related to age-associated changes in social relationships. To do this, we formed novel flocks of either young adult (6 mo – 1 y) or older adult (≥3 y) male budgerigars and tracked their contact call sharing patterns and social dynamics over time. Vocal learning can be measured in a number of ways; in this study, we characterize three components of vocal learning that each capture different aspects of this multidimensional trait. We measure *vocal diversity*, or the size of an individual's call repertoire; *vocal plasticity*, or the change in acoustic similarity between an individual's call repertoire over time; and *vocal convergence*, or the acoustic similarity of an individual's call repertoire to that of its flockmates. Likewise, sociality can be quantified in numerous ways; here we focus on three dimensions of social behaviour (*inter-individual proximity*, *affiliation*, *agonism*) and use social network analysis to compute the strength of social relationships and the degree of connectedness among flockmates, or density, with respect to each social dimension. We hypothesized that vocal learning exhibits senescence, as is typical of complex cognitive traits. If true, we predicted that older adults will exhibit less vocal plasticity and less contact call convergence compared to young adults. Additionally, we hypothesized that aged individuals exhibit greater social selectivity and a positivity bias when integrating into a social group. If true, we predicted that flocks of older adults will have less connected social networks in all three social dimensions. Additionally, we predict that flocks of older adults will exhibit stronger affiliative ties and weaker agonistic ties than flocks of young adults.

## 2. Methods

### (a) Animal housing and care

We acquired 48 adult male budgerigars from a commercial breeder (McDonald Bird Farms, Kerrville, TX; *N* = 42) and from our own research colony (*N* = 6). These individuals were classified as either young adults (6 mo – 1 y; *N* = 24 from breeder) or older adults (≥ 3 y; *N* = 18 from breeder and *N* = 6 from colony) based on known hatch dates from our colony or approximate ages provided by the breeder. The commercial breeder provided young birds housed in four separate aviary buildings and old birds housed in three separate aviary buildings. Together with a set of old males from our colony, this created four independent source populations for each age class, wherein members belonging to different sources were unfamiliar to each other, allowing us to combine individuals from separate sources to form flocks with novel membership.

**Figure 1.**
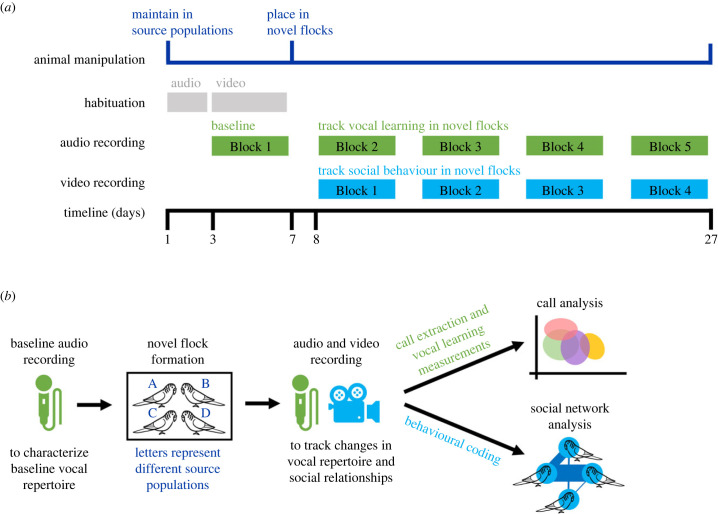
Experimental design. (*a*) Experimental timeline outlining timing of flock manipulations and recording of vocal and social behaviour. (*b*) Experimental overview including data collection and analysis procedures.

Prior to such flock formation, these independent source populations were housed in four separate rooms at the New Mexico State University Animal Care Facility (ACF), with each room containing one young source and one old source housed in separate cages, except for one room in which birds of both ages were kept in the same large cage. Birds were maintained on a 12/12 light/dark cycle and fed a commercial seed diet and vitamin-supplemented water ad libitum. The sex of all individuals was confirmed to be male via PCR genetic sexing (using the P0, P2 and P8 avian primers [32]) on blood samples collected prior to beginning the experiment following procedures in Pease *et al*. [33]. Budgerigars were habituated to the experimental conditions by placing them in sound chambers and by attaching a video camera atop their cage for two 50 min sessions per bird per habituation method. Audio habituation sessions took place on days 1–2 of the experimental timeline and video habituation sessions took place on days 3–6 (figure 1*a*). Experimental cages (79 × 52 × 102 cm), used to house novel flocks, consisted of an identical layout of food dishes, water and perches. One young and one old flock were housed in separate experimental cages in each experimental room in ACF. Flocks were visually isolated from one another using opaque black cloth barriers on the cage sides, since prior studies indicate a lack of visual interaction diminishes call sharing between separate social groups [14]. All procedures conducted in this study were approved by the New Mexico State University Animal Care and Use Committee (protocol number 2020-030).

### (b) Experimental outline

Prior to the formation of novel flocks, we collected each individual's initial call repertoire by recording its vocalizations while it was still housed in its source population. Birds were audio-recorded on days 3–6 of the experimental timeline (figure 1*a*) according to the audio recording protocol described below. Calls collected during this first recording block are considered birds' baseline vocal repertoires and were used to compare later call repertoires to track changes in repertoire diversity and changes in acoustic structure. On day 7, we formed flocks composed of four birds belonging to the same age class that were randomly selected from separate source populations and allowed birds to habituate to their new flockmates for one day. Birds were then audio- and video-recorded for 20 days (days 8–27) to track vocal learning from and social integration with these novel flockmates; previous literature shows that adult male budgerigars can imitate the calls of flockmates within one week [14]. Recordings were conducted in four 4-day blocks; each block was followed by a break day during which birds were generally undisturbed besides routine care; however, on several occasions, break days were used to re-record audio and/or video to compensate for technical issues occurring in the previous recording block. This entire experimental timeline was repeated for three rounds, with two flocks per age class formed per round yielding six replicate novel flocks per age class overall. This experiment was conducted beginning on 14 December 2020 and ending on 27 April 2021.

### (c) Audio recording

Audio recordings were collected between 07.00 and 13.00, during which calls of each bird were recorded in daily 55-min sessions, yielding a total of 4 sessions per bird prior to novel flock formation and 16 sessions per bird following novel flock formation. The order in which birds were recorded was rotated so that each individual was recorded first once in each recording block. During each audio recording session, one focal bird from each social group was isolated from its flockmates and individually recorded in a small wire cage (15 × 10.5 × 19 cm) placed inside a custom-built sound-isolation chamber (60 × 34 × 32 cm) containing a microphone (Audio-Technica Pro 37). The acoustic chamber was a cooler (Igloo, Shelton, CT) lined with acoustic foam and had a transparent plexiglass door to allow focal individuals and nearby flockmates to see each other. The chamber was placed 80 cm from the home cage so that the focal bird was in visual but not acoustic contact with its social associates—this distance separation procedure is a common method of eliciting contact calls in this species [14,28]. Recordings were processed using a Focusrite Saffire Pro digitizer, directly saved to a Dell computer using Sound Analysis Pro software with a sampling rate of 44.1 kHz.

### (d) Video recording

After the formation of novel flocks, video recordings were collected in two 80-minute sessions per flock per recording block, during which social interactions between novel flockmates were recorded, yielding a total of 8 sessions per flock. One early morning session (07.00–09.00) and one late morning session (11.00–13.00) were conducted per flock per recording block. At the time of recording, a camera (Dragon Touch Vision 3 Action Camera) was affixed onto the top of the cage using a gooseneck mount (Lamicall Flexible Arm) and manually positioned so that the entire cage was in view. Videos obtained during early morning sessions (the time period during which pilot data showed birds were most active) were coded for affiliative behaviours (allopreen, allofeed, kiss and head bob) and agonistic behaviours (bill thrust, kick and rejected affiliative interaction) using continuous behavioural sampling (example videos of each coded behaviour can be found in the Dryad dataset). Sampling started 30 min after the experimenter left the room, since birds often remained still for period of time after this disruption, and was conducted for a duration of 40 min. These behaviours were defined in an ethogram developed based on previous descriptions of budgerigar social behaviour [34–36].

Videos from both early and late morning recording sessions were coded for inter-individual proximity. Snapshots were taken and saved as TIFF image files (VLC Media Player 3.0.12 Vetinari) at 4-minute intervals starting from 30 min after the experimenter left the room and for a duration of 40 min, yielding 11 snapshots per flock for each recording session (88 snapshots overall per flock). The line tool in Image J 1.53e was used to mark the distance between each pair of birds in the flock, with lines originating at the top of each bird's head at eye level [37]. Then, an in-frame ruler (cage perch marked every 5 cm in Sharpie) was used as a scale for measuring these inter-individual distances.

### (e) Call analysis and statistics

Sound files were first downsampled from 44.1 kHz to 19 kHz to improve computational efficiency. Contact calls were selected from original recordings in R v. 4.0.5 [38] by first using the ‘optimize_energy_detector’ function in the package *ohun* (v. 0.0.1; [39]) to apply optimized thresholds for sensitive detection of calls based on amplitude, frequency range, and duration parameters. Spectrograms were generated for a subset of these detections using the ‘spectrograms’ function in *warbleR* (v. 1.1.27; [40]) and then visually sorted as either ‘signal’ (contact calls) or ‘noise’ (cage noise, feather ruffling and other vocalization types). Sorting detections in this way created a training dataset for implementing supervised random forests, a machine learning algorithm used as a categorical classification approach with growing popularity in the classification of animal vocalizations [41–43]. The random forest model was trained to predict contact calls based on these manually sorted detections using the R package *ranger* (v. 0.12.1; [44]).

In addition to contact calls, budgerigars also produce a complex, highly variable and multi-syllabic song called warble, which contains elements that resemble contact calls in structure but differ in behavioural context [45]. Due to this high acoustic similarity, the random forest model included these contact call-like warble elements in its prediction of calls, requiring a quality control step in which annotations from predicted calls (formatted using the R package *Rraven*; [46]) were visually inspected in Raven Pro v. 1.6 [47] so that warble elements could be removed, yielding a total of 49 573 confirmed contact calls out of the 150 534 predicted calls.

Seventeen acoustic features—including various frequency parameters, duration, entropy, skew and kurtosis—were extracted and scaled (i.e. zero-centered with unit variance) for each contact call using the *warbleR* ‘spectro_analysis’ function with a 150-sample window length and a 0.5–8.0 kHz bandpass filter. We reduced the dimensionality of these extracted acoustic measures using t-Distributed Stochastic Neighbor Embedding (t-SNE) [48] with the R package *Rtsne* (v. 0.15). The first two dimensions were plotted to generate an acoustic trait space wherein acoustically similar calls appear closer together. We then used the R package *PhenotypeSpace* (v. 0.1.0; [49]) to quantify acoustic areas to assess the effect of age on an individual's acoustic repertoire over time. To characterize the acoustic space of each individual per audio recording block, we computed kernel density areas for calls subset by individual identity and time, which quantifies irregular areas with higher precision [50].

Three vocal learning measures were then extracted from these acoustic spaces. First, change in *vocal diversity* was estimated as the change in area of an individual's acoustic space compared to its own baseline area. Specifically, we subtracted an individual's total acoustic area during the first recording block from that individual's acoustic area in each of the four subsequent recording blocks. Second, *vocal plasticity* was defined as the amount of acoustic similarity between an individual's acoustic space and its acoustic space in block 1, where less similarity to self indicates greater vocal plasticity. Acoustic similarity here was calculated as the proportion of acoustic space area that overlaps between an individual's acoustic space in block 1 and its acoustic space in each of the four subsequent recording blocks, where higher values indicate more similarity. We calculated a single pairwise mean overlap for each comparison since the proportion of space A that overlaps space B and the proportion of space B that overlaps space A are two separate values. Additionally, this computation accounts for irregular densities of acoustic spaces, such that overlap in high-density areas have higher similarity values compared to overlap in lower-density areas. Finally, *vocal convergence* was defined as the amount of acoustic similarity between an individual's acoustic space and the combined acoustic space of its three flockmates. This was also calculated using pairwise mean overlap.

Generalized linear mixed-effects models (GLMMs) were used to determine whether adult age affects *vocal output*, the number of contact calls produced and the three previously defined vocal learning measures. Models were created separately for each vocal response variable (vocal output, vocal diversity, vocal plasticity and vocal convergence), fitting an appropriate distribution for each data type. Adult age, recording block (modelled as a continuous factor) and their interaction were used as fixed effects. Individual identity and experimental flock were included as random effects to account for non-independence arising from repeated measures for each individual across recording blocks and the nesting of multiple individuals within each replicate flock, respectively. Vocal output was modelled using a negative binomial error distribution and log link function for counts, using the *lme4* package (version 1.1-27.1; [51]). Vocal diversity was modelled with a Gaussian error distribution using the *lme4* package. Vocal diversity and vocal convergence, both proportional data based on overlaps of acoustic spaces, were modelled with a beta error distribution using the *glmmTMB* package (v. 1.1.4; [52]). The interaction term was dropped from the full models if it was nonsignificant.

### (f) Social network analysis and statistics

We used a social network approach to determine age-related variation in social integration wherein social structure is characterized by nodes, representing individuals, connected by edges, representing existing relationships [53,54]. Social networks for each flock were constructed for each of the three social dimensions of interest—proximity, affiliation and agonism—using the R package *igraph* (version 1.2.6; [55]). Adjacency 4 × 4 matrices were created for each flock, with cells containing the edge value representing the interactions observed between each dyad. Edges of proximity network graphs were weighted by the proportion of observations in which pairs of flockmates were in ‘close proximity’, a threshold we set as ≤10 cm, equivalent to one budgerigar body length or the distance at which direct physical interaction is possible. Proportions rather than counts were used to deal with different total numbers of observations across individuals arising from birds being absent from the focal plane at the sampling time (such as being perched on the cage wall or on the floor of the cage), which precluded accurate distance measurements in ImageJ. Affiliative and agonistic network graphs were weighted by the frequency of affiliative and agonistic interactions, respectively, that were observed to have occurred between pairs of flockmates during the sampling period.

Networks were made for close proximity occurrences and interactions summed across the entire video-recording period as well as interactions subset by the first (blocks 1 & 2) and second (blocks 3 & 4) halves of the recording period for a separate analysis of whether age influences how social relationships change over time (see electronic supplemental material). From these networks, we calculated network density and individual strength (or weighted degree) [56]. Density is a measure of group-level connectivity, calculated by dividing the number of observed edges in a network by the total number of possible dyadic connections. Strength is a measure of individual gregariousness, calculated as the sum of edge weights connected to a node.

All statistical analyses of social network metrics were carried out in R v. 4.0.5 [38]. To deal with non-normally distributed data, we used generalized linear models (GLMs) to determine whether density and strength can be predicted by adult age class using the *lme4* (version 1.1-27.1) and *MASS* (version 7.3-58.1; [57]) packages in R. Models were created separately for each network type (proximity, affiliative, agonistic). For density, we fitted a GLM with a binomial error distribution and a logit link function, for proportional data based on counts, and included age class as a fixed effect. For strength, we fit GLMMs with a negative binomial error distribution and log link function for count-based affiliative and agonistic interaction frequencies and a gamma error distribution and inverse link function for continuous, right-skewed proximity proportions. For these models of strength as a response variable, we included age class as a fixed effect and experimental flock as a random effect to account for non-independence arising from multiple individuals belonging to the same social unit.

## 3. Results

### (a) Effect of age on vocal learning

**Figure 2.**
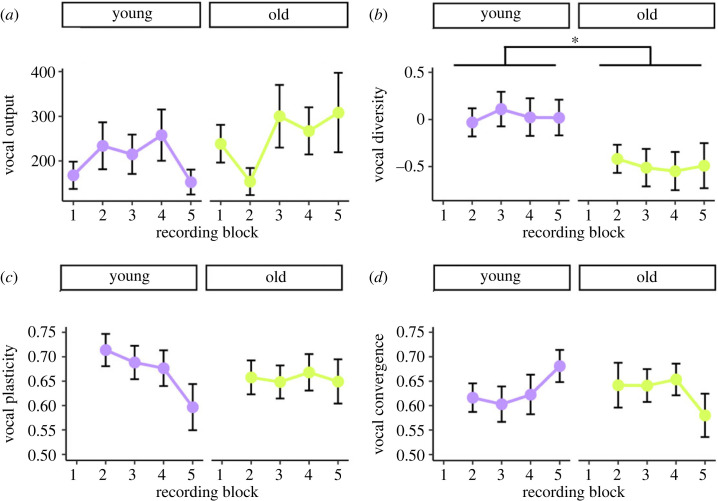
Change in vocal output (*a*) and vocal learning measures (*b–d*) over time by adult age class presented as mean ± s.e. plots. *p < 0.05. Vocal plasticity, measured as similarity to starting self, and vocal convergence both exhibit significant interactions between age and recording block, as detailed in table 1.

On average, 193 ± 19 (s.e.) contact calls were collected per individual in each recording block from young adults and 234 ± 25 from older adults. Various contact call acoustic parameters were found to differ with age; calls produced by older adults were notably characterized as shorter in duration, higher in frequency and lower in frequency modulation (see electronic supplementary material, figure S1). Vocal output did not significantly differ between young and older adults (*z* = −0.05, *p* = 0.957, figure 2, table 1). Of the three vocal learning measures, only change in vocal diversity was significantly predicted by adult age (*t* = 2.33, *p* = 0.025). Mean change in acoustic area from recording block 1 to each of the subsequent blocks was 0.030 ± 0.089 (s.e.) for young adults and −0.491 ± 0.097 for older adults. While young adults exhibit a mean increase in acoustic area, indicative of a more acoustically diverse contact call repertoire in response to joining a novel flock, this change is close to 0, suggesting that vocal diversity remained at least stable in young adults. The mean decrease in acoustic area exhibited by older adults, however, indicates a reduction in vocal diversity over the course of the experiment (see electronic supplementary material, figure S2 for a sample of an individual exhibiting an increase in vocal diversity, typical of young adults and an individual exhibiting a decrease in vocal diversity, typical of older adults).

**Table 1.** Generalized linear mixed modelling output to determine whether adult age predicts vocal output and three vocal learning measures and whether young and older adults differ in how their vocal responses change over time, with recording block modelled as a continuous variable. A separate model was specified for each response variable with individual identity and flock as random effects. Significant relationships are shown in bold.

response	fixed effect	estimate	s.e.	critical value	*p*-value
vocal output	age	0.134	0.379	*z* = 0.354	0.723
	recording block	−0.070	0.055	*z* = −1.283	0.199
vocal diversity	**age**	**0**.**507**	**0**.**218**	***t* = 2.325**	**0**.**025**
	recording block	−0.001	0.036	*t* = −0.033	0.974
vocal plasticity	age	0.467	0.213	*z* = 1.558	0.119
	recording block	−0.004	0.039	*z* = −0.103	0.918
	**age × recording block**	**−0**.**166**	**0**.**054**	***z* = −3.073**	**0**.**002**
vocal convergence	age	−0.407	0.254	*z* = −1.602	0.109
	recording block	−0.068	0.057	*z* = −1.192	0.234
	**age × recording block**	**0**.**162**	**0**.**079**	***z* = 2.053**	**0**.**040**

No significant differences were observed between young and older adults in overall levels of vocal plasticity or vocal convergence relative to baseline levels, suggesting that these aspects of vocal learning are not dependent on adult age (figure 2 and table 1). Adult age and recording block, however, did interact to predict these two measures (table 1). Overlap with starting self appears to decrease over time within young adults and remain stable over time in older adults (figure 2). Acoustic overlap with the contact call repertoire of one's flockmates appears to increase over time for young adults and to decrease over time for older adults, with the greatest amount of change occurring between blocks 4 and 5 (figure 2).

### (b) Effect of age on social integration

**Figure 3.**
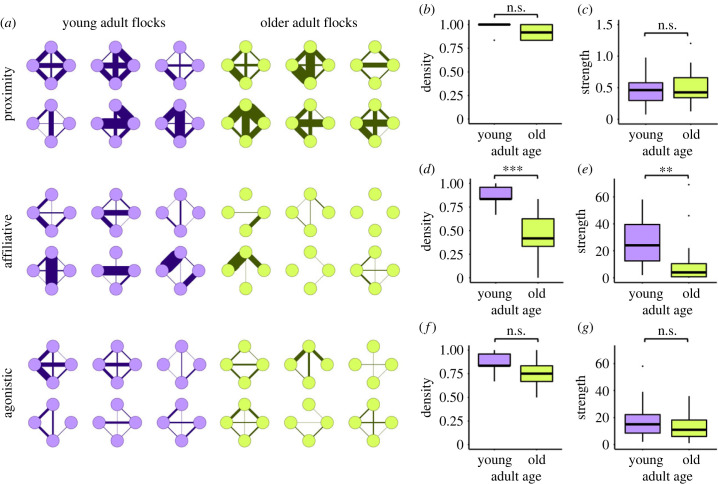
Social networks and quantification of social integration by adult age class across the entire video-recording period. (*a*) Social network graphs for *N* = 6 replicate flocks per adult age class, depicting relationships between flockmates based on proximity, affiliation and agonism in separate rows. Nodes represent individual birds (*N* = 4 per flock) and the thickness of the lines (edges) connecting nodes represents either the proportion of observations during which pairs were in close proximity (row 1) or the frequency of affiliative or agonistic interactions observed (rows 2–3). Individuals maintain the same node position across the three network types. (*b–g*) Boxplots represent social network metrics computed for proximity (*b*–*c*), affiliation (*d–e*) and agonism (*f–g*). Colour is used to represent adult age class, where young adults are in purple and older adults in green. ***p < 0.001; **p < 0.01; n.s. = not significant.

In general, young adults had higher social activity and more positive behaviours than older adults (table 2). From social networks constructed across the entire video-recording period for each social dimension (figure 3*a*), adult age was only found to significantly influence social integration for affiliative interactions. Flocks of young adults have a higher affiliative density (*z* = 3.487, *p* < 0.001), and thus are more affiliatively connected compared to flocks of older adults (figure 3*d*). This means that older adults are directing affiliative interactions to a smaller proportion of potential social partners compared to young adults. Affiliative connections among young individuals were also stronger compared to older birds (*z* = 2.623, *p* = 0.009), meaning that young adults exhibited higher numbers of repeated affiliative interactions (figure 3*e*). Age did not significantly affect agonistic density (*z* = 1.177, *p* = 0.239) and strength (*z* = 0.881, *p* = 0.379) (figure 3*f,g*) or proximity-based density (*z* = 0.981, *p* = 0.327) and strength (*t* = −0.523, *p* = 0.612) (figure 3*b,c*).

**Table 2.** Summary of social interactions. Young adults tend to exhibit higher social activity and a bias towards more positive behaviours while older adults engage in affiliative and agonistic behaviours at more similar proportions.

age	affiliative interactions	agonistic interactions	affiliative interactions per flock (mean ± s.e.)	agonistic interactions per flock (mean ± s.e.)	total
young	316 (61%)	202 (39%)	53 ± 9	34 ± 9	518
old	116 (46.4%)	134 (53.6%)	19 ± 10	22 ± 6	250

## 4. Discussion

In this study, we investigated whether vocal learning diminishes with advanced age in an open-ended learner and how this socially mediated trait might be related to age-related changes in social relationships. We formed novel flocks of male budgerigars of two adult age classes, young adults within their first year of age and older adults in at least their third year of age, and tracked their contact call repertoires as well as affiliative-, agonistic- and proximity-based social associations over time. We found that older adults exhibited a significant reduction in contact call vocal diversity and had weaker and less dense affiliative social associations compared to young adults. Overall levels of vocal plasticity and vocal convergence, however, were similar between these two age classes, suggesting the maintenance of these two components of vocal learning ability into late adulthood, despite weaker affiliative social bonds. Below we contrast these patterns in contact call acoustics, vocal learning and social integration between young and older adult budgerigars and discuss the implications for cognitive and social senescence across taxa.

### (a) Social ageing patterns and vocal diversity

Our social network results largely parallel the social ageing phenotype identified in past studies. Overall, we observed fewer social interactions among older budgerigars, similar to patterns reported in ageing humans and non-human primates [5,20,22]. Contrary to the positivity bias associated with ageing in other species, it was young adult budgerigars, not older adults, that exhibited a bias towards friendlier behaviours, with most of their total interactions being affiliative compared to more equal proportions of affiliative and agonistic behaviours in older adults. Similar to the one other study of social relations and ageing in a parrot species, we found stronger social associations in younger individuals but, in contrast to this study, we found an age-related difference in the number of social associations, with older adults forming fewer affiliative relationships [23].

Older adult budgerigars exhibited a decrease in vocal diversity as they had smaller acoustic space areas after being placed in their novel flocks, while young adults exhibited a slight increase in their vocal diversity. This reduction in the diversity of older adults' contact call repertoire is consistent with findings by Pavlova *et al*. [18] of reduced song repertoire size in older European starlings, but contrasted with findings of increased song repertoire size with old age in collared flycatchers (*Ficedula albicollis*) [58]. The reduction in vocal diversity we observed in older budgerigars suggests diminished learning ability with respect to one vocal measure. Other aspects of vocal learning, however, can be considered to be largely maintained since older adults still achieved similar overall levels of vocal plasticity and vocal convergence to young adults.

Since budgerigars use contact calls to mediate both dyadic and group-level social relationships, a reduction in contact call repertoire diversity may be directly related to our finding of lower affiliation among older adults compared to young adults. It may be the case that older individuals’ selectively interacting with fewer social partners relaxes their need to have a diverse contact call repertoire and may be indicative of a general pattern of increased social selectivity with ageing [21,22]. Our finding of not only fewer but also weaker affiliative connections among older adults further suggests a link between contact call repertoire and affiliation.

### (b) Vocal plasticity and vocal convergence

Since social bonding is thought to be facilitated by contact call sharing [19,59], fewer and weaker affiliative social relationships among older adults might also be reflected by reduced vocal convergence. We did not, however, observe any significant differences between young and older adults in acoustic overlap with the contact calls of flockmates. This may be because we measured contact call convergence by comparing the acoustic overlap of an individual's acoustic space with the combined acoustic spaces of its other three flockmates, while we characterized social relationships based on dyadic interaction frequencies.

The equivalent amounts of vocal convergence with flockmates that we observed between young adult and older adult budgerigars suggest that the open-ended vocal learning program, with respect to acoustically matching the calls of social associates, may be resilient to ageing in this species. Older adults also did not differ from young adults with respect to vocal plasticity, with both age groups ending with comparably reduced levels of acoustic similarity to their baseline contact call repertoires. Thus, we find no evidence of the predicted age-related cognitive declines typically associated with senescence with respect to vocal convergence and vocal plasticity, two key components of vocal learning. The maintenance of these two components is in line with recent work on senescence in teleost fishes that found no evidence of age-related declines in multiple physiological systems and highlighted the wide variation in rates of senescence among vertebrates [60]. We note that our findings of a lack of deterioration in vocal plasticity and vocal convergence should be interpreted with some caution as our older adult age class may not capture very late adulthood, given that some budgerigars in captivity have been known to live many years beyond the mean life expectancy of 4.57 years [30,31]. It does appear however that birds close in age to the mean life expectancy of this species seem to maintain similar levels of vocal plasticity and vocal convergence to young adults. Our work opens avenues for exploring the neural mechanisms that underly this apparent resilience to senescence in vocal learning ability.

## Data Availability

The supporting datasets and code have been uploaded to Dryad (https://doi.org/10.5061/dryad.fttdz08xr [61]). Supplementary methods and results are provided in electronic supplementary material [62].
